# Nearly complete genome sequence of one GII.17 Norovirus identified by direct sequencing from HuZhou, China

**DOI:** 10.1002/mgg3.446

**Published:** 2018-07-10

**Authors:** Lei Ji, Liping Chen, Deshun Xu, Xiaofang Wu, Jiankang Han

**Affiliations:** ^1^ Huzhou Center for Disease Control and Prevention Huzhou China

**Keywords:** complete genome, genetic analysis, GII.17, norovirus

## Abstract

**Background:**

Human norovirus is the leading cause of acute gastroenteritis worldwide. However, in vitro culture system is complicated for human norovirus. Sequence analysis became more useful for norovirus research, particularly when using complete genomic sequences.

**Methods:**

Real‐time RT‐PCR (qPCR) was performed for norovirus detection. Three modified paris of PCR primes were designed based on the alignment of the novel GII.17 norovirus complete sequence available in Genbank., which could amplify three overlapping fragments cover the whole genome. The PCR fragments were sequencing by Sanger sequence with Primer walking methods. Genogroup and genotype were assigned using the Norovirus Noronet typing tool and the strains were named according to the time of isolation. The phylogenetic analysis was conducted using MEGA software (ver. 6.06).

**Results:**

One nearly complete genome sequence were obtained from sample collected from Huzhou, China. The partial genome sequence of the HuzhouNS2014603 strain is composed of 7556 nucleotides (nt).The strain was classified as GII.17 genotype both in ORF1 and ORF2, and was most closely related to the LC037415.1/Hu/GII.17/Kawasaki308 strain. Within the GII.17 cluster, the 2013/14 season strains were grouped separately from the GII.17 strains detected in 2014/15. HuzhouNS2014603 was clustered with the 2014/15 season strains. Compared with other strains selected, there are 98 variable residues across the VP1 domain. Among the 98 variable amino acids, 13 (13.3%) were observed in the shell domain and 22 (22.4%) in the P1domain; most of the substitutions and insertions were located in the P2 domain, account for 63 (64.3%).

**Conclusions:**

This is the first report of the nearly complete genome of the novel GII.17 by direct sequencing method in the Huzhou area. The results of this study could be helpful for the study of the genetic evolution of the virus, the development of rapid diagnostic reagents and the design of vaccine.

## 1 BACKGROUND

Human norovirus is the leading cause of acute gastroenteritis worldwide (Ahmed et al., 2014). This highly infectious virus can be transmitted by various modes, such as direct/indirect contact, waterborne transmission, foodborne transmission, and even airborne transmission in certain settings (Kroneman et al., 2008; Said, Perl, & Sears, 2008).

The genome of norovirus is a single‐stranded positive‐sense RNA approximately 7.7 kb in length that is covalently linked to VPg at the 5′end and polyadenylated at the 3′end. The genome is organized into three open reading frames (ORFs): ORF1 encodes for a polyprotein required for replication such as NTPase, protease, and RNA‐dependent RNA polymerase (RdRp); ORF2 encodes the viral protein 1 (VP1); and ORF3 encodes the viral protein 2 (VP2) (Hardy, 2005). The large genetic divergence of human norovirus results in two genogroups and many genotypes (Green, 2013; Robilotti, Deresinski, & Pinsky, 2015; Vinjé, 2015). GII strains, mostly GII.4, were responsible for 75%–100% of norovirus cases worldwide for the last decades (Kumazaki & Usuku, 2015; Patel et al., 2008; Zheng, Widdowson, Glass, & Vinje, 2010). In the winter of 2014–2015, a novel variant of norovirus GII.17 emerged and became predominant in Huzhou, and steadily replaced the previously circulating GII.4 Sydney 2012 strain (Han et al., 2015).

Here, we report the nearly complete nucleotide sequence of a novel HuzhouNS2014603 strain belonging to GII.17 Kawasaki 2014 in Huzhou, Zhejiang Province, China. Phylogenetic and evolutionary relationships analysis with other related viral strains circulating worldwide at different time periods were performed.

## 2 METHODS

### 2.1 Specimen collection

In a norovirus surveillance study conducted in patients with sporadic gastroenteritis at the First People's Hospital in Huzhou, a novel GII.17 variant was identified based on partial RNA‐dependent RNA polymerase region (RdRp) and capsid protein (VP1) gene. This study was part of the regional norovirus gastroenteritis surveillance program conducted.

The definition of acute gastroenteritis was diarrhea (≥3 loose stools within a 24‐hr period), possibly accompanied by vomiting, abdominal pain, fever, and nausea. All stool samples were freshly collected with a sterile container and sent to Huzhou Center for Disease Control and Prevention for immediate storage at −70°C prior to analysis.

### 2.2 Viral RNA extraction and norovirus detection

Viral RNA was extracted from 140 μl of supernatant of a 10% (w/v) fecal suspension using a QIAamp Viral RNA Mini Kit (QIAGEN, Hilden, Germany) according to the manufacturer's protocol. RNA extracts were subjected to the reverse transcription polymerase chain reaction (RT‐PCR) or stored at −70°C until further use. Genogroup‐specific primers and probes described previously were used to detect norovirus by real‐time RT‐PCR (qPCR) (Jothikumar et al., 2005). Primer and probe sets JJV1F/JJV1R/JJV1P and JJV2F/COG2R/RING2‐TP were used to screen for GI and GII norovirus strains respectively. RT‐qPCR was carried out using a One Step PrimeScript RT‐PCR Kit (DRR064; TaKaRa, Dalian, China). Amplification conditions were described previously (Ji et al., 2013).

For genotyping, the primer set JV12Y/JV13I was used to amplify the 3′‐end of the RdRp (RNA‐dependent RNA polymerase) gene (region A in ORF1) (Vennema, de Bruin, & Koopmans, 2002). Primer sets G1SKF/G1SKR and G2SKF/G2SKR were used to amplify the 5′‐end of the capsid protein (VP1) gene (region C in ORF2) for GI and GII respectively (Kojima et al., 2002). RT‐PCR was carried out using a One Step RNA PCR Kit (TaKaRa) with the amplification conditions described previously (Ji et al., 2013). After amplification, 5** **μl of the PCR products was visualized by agarose gel electrophoresis. The residual PCR products were purified using a QIAquick PCR purification kit (Qiagen, Leusden, The Netherlands), and the purified products were sequenced directly at both ends with amplification primers by Genscript Biotechnology (Jiangsu, China).

### 2.3 Full‐length genome amplification and sequencing

The nearly full‐length genome of HuzhouNS2014603 strain was amplified with three pairs of primer sets modified from two papers based on the complete genome sequences of the Novel GII.17 norovirus (Chan et al., 2014; Cotten et al., 2014) (Table 1). To facilitate the sequencing, the first two fragments were obtained by one‐step reverse transcription PCR (RT‐PCR) (TAKARA, Japan), following the manufacturer's protocol with the amplification conditions as follows: 42°C for 30 min, 98°C for 30 s, followed by 35 cycles of 98°C for 10 s, 64°C for 20 s, and 72°C for 1.5 min, and a final step at 72°C for 5 min. Conversion of viral RNA to cDNA was performed using SuperScript III reverse transcriptase (Thermo Scientific) with a tagged oligo dT primer (RT primer) according to the manufacturer's protocol before amplify the third fragment. The RT‐PCR was carried with the following cycling profile: 98°C for 30 s, followed by 35 cycles of 98°C for 10 s, 64°C for 20 s, and 72°C for 1.5 min, and a final step at 72°C for 5 min. After amplification, 5 μl of the PCR products was visualized by agarose gel electrophoresis. The residual PCR products were purified using a QIAquick PCR purification kit (Qiagen, Leusden, The Netherlands), and the purified products were sequenced directly at both ends with amplification primers by Genscript Biotechnology (Jiangsu, China).

**Table 1:** Primers used for norovirus full‐length genome amplification Sequence location is based on the sequence of KT380915.1

Primer	Sequence(5′–3′)	Length	Location	Reference
RT primer	GACTGACTAGCTATCGGAGCATCG(T)31			
No17‐S1‐F1	GTGAATGAAGATGGCGTCTAAC	22	1–22	KT380915
No17‐S1‐R1	GCTGTATGTTTCTTGCCCCG	20	2633–2652	
No17‐S2‐F1	AAACCTCTGGTCGAAGCCAC	20	2521–2540	
No17‐S2‐R1	TATTTTGGCCAGTGACGGGT	20	5210–5229	
No17‐S3‐F1	TGTGAATGAAGATGGCGTCG	20	5002–5022	
No17‐S3‐R1	GACTGACTAGCTATCGGAGCATCG	24		

### 2.4 Sequence analysis and phylogenetic analysis

Genotypes were determined using the online norovirus Typing Tool (http://www.rivm.nl/mpf/norovirus/typingtool) (Kroneman et al., 2011), and the strains were named according to the isolated place, time, and sample number. A phylogenetic tree was generated using the neighbor‐joining method with MEGA software (ver. 6.06) (Tamura, Stecher, Peterson, Filipski, & Kumar, 2013). The evolutionary distance was calculated based on the maximum composite likelihood model, and the reliability of each branch was assessed with 1,000 bootstrap replicates. Similarity searches were carried out using the BLAST search (http://www.ncbi.nlm.nih.gov/BLAST/) utility of the National Center for Biotechnology Information (NCBI) database. ORF positions were verified by the ORF finder of NCBI and compared with the reference sequence. The nearly full‐length genome sequences of norovirus strains were aligned, and phylogenetic analysis was performed with other published reference strains obtained from GenBank database (http://www.ncbi.nlm.nih.gov/genbank) using the DNAStar program. The VP1sequences were compared between HuzhouNS2014603 and the strains in the same cluster at the nucleotide and amino acid levels.

### 2.5 Accession numbers of references norovirus strains

The accession numbers of norovirus strains used as references for phylogenetic analysis are as follows: L07417.1, AB039780.1, EU424333.1, AB039777.1, AB074893.1, AY823305.2, AB083780.1, AB220922.1, X86557.1, U07611.2, AY772730.1, AF397156.1, DQ456824.1, KJ196284.1, EU921354.2, AB039782.1, LC043139.1, LC043305.1, LC043168.1, KU561250.1, AB983218.1, KJ156329.1, LC043167.1, KP998539.1, KT346356.1, KR083017.1, LC037415.1, KT970371.1, KR270448.1, KR270446.1, KT253245.1, KT380915.1, KU561256.1, DQ438972.1, GQ266697.1, GQ266696.1, KP698930.1, KP864103.1, KR020503.1, KP864102.1, KU561252.1, KT780402.1, KU757050.1, KU561248.1, KU561254.1, KU561255.1, KU561253.1, AY502009.1, KJ196228.1, JN699043.1, KC597139.1, KJ196286.1

## 3 RESULTS

### 3.1 The nearly complete genome of the HuzhouNS2014603 strain

The nearly complete genome sequence of the HuzhouNS2014603 was submitted to GenBank with accession number MG692610.1. The sequence was obtained by sequence assembly with nearly full length of 7556 nucleotides (nt). The 5′ and 3′ ends of the genome have 4nt and 61nt noncoding regions. The sequence of the coding region of 7490nt is divided into three ORFs, ORF1, ORF2, and ORF3 of 5,109, 1,623, and 780 nt in length respectively. ORF1 and ORF2 had an overlap of 20 nucleotides (nt 5,094–5,113), whereas ORF2 and ORF3 had a single nucleotide overlap (A at position 6716). GII.P17_GII.17/KR/2015/CAU‐267 (GenBank accession: KU561254.1) showed the highest query cover and percent nucleotide identity with our sequence based on the whole genome.

### 3.2 Phylogenetic analysis of the HuzhouNS2014603 genome

**Figure 1:**
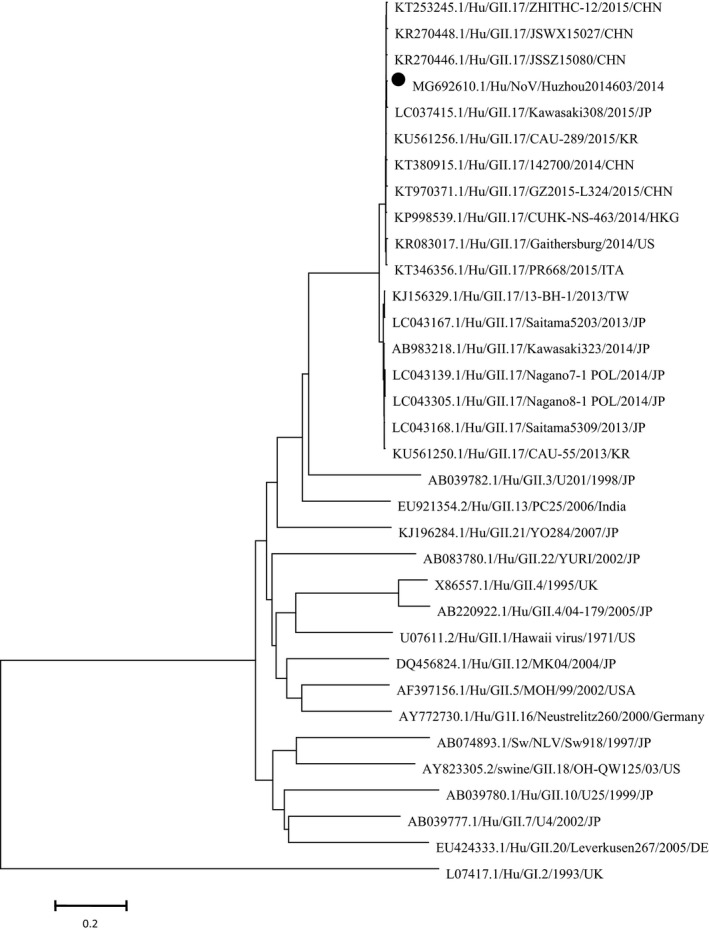
Phylogenetic tree based on the nearly full‐length nucleotide sequence of representative strains for each NoV genotype of GII strains. HuzhouNS2014603 is highlighted with a black dot. The accession numbers of norovirus strains used are as follows: L07417.1, AB039780.1, EU424333.1, AB039777.1, AB074893.1, AY823305.2, AB083780.1, AB220922.1, X86557.1, U07611.2, AY772730.1, AF397156.1, DQ456824.1, KJ196284.1, EU921354.2, AB039782.1, LC043139.1, LC043305.1, LC043168.1, KU561250.1, AB983218.1, KJ156329.1, LC043167.1, KP998539.1, KT346356.1, KR083017.1, LC037415.1, KT970371.1, KR270448.1, KR270446.1, KT253245.1, KT380915.1, KU561256.1

**Figure 2:**
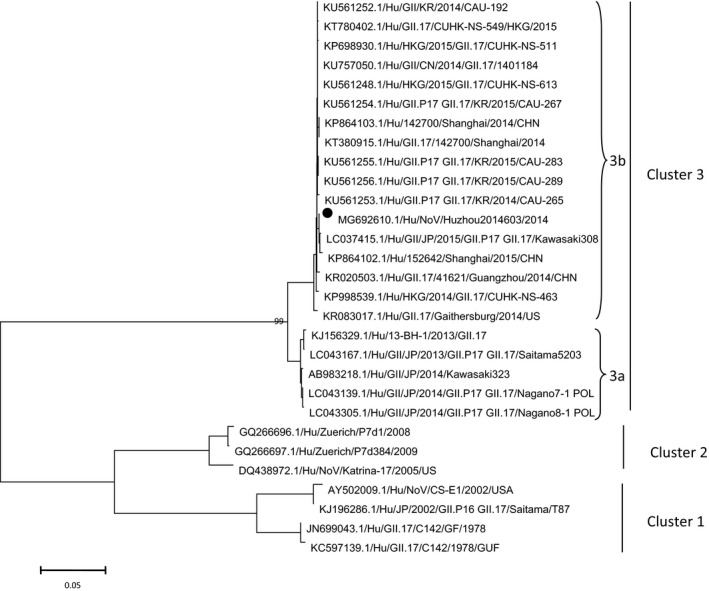
Phylogenetic tree based on the full‐length nucleotide sequence of capsid protein of the NoV GII.17 genotype. HuzhouNS2014603 is highlighted with a black dot. The accession numbers of norovirus strains used are as follows: DQ438972.1, GQ266697.1, GQ266696.1, LC043139.1, LC043305.1, KJ156329.1, LC043167.1, KR083017.1, KP698930.1, KP998539.1, KP864103.1, KR020503.1, LC037415.1, KP864102.1, KU561252.1, KT780402.1, KU757050.1, KU561248.1, KU561254.1, KT380915.1, KU561255.1, KU561256.1, KU561253.1, AB983218.1, AY502009.1, KJ196228.1, JN699043.1, KC597139.1

To understand the genetic relationships between HuzhouNS2014603 and other completely sequenced GII norovirus, phylogenetic analysis based on the complete genome with representative strains was performed. The HuzhouNS2014603 strain was classified as GII.17 genotype both in ORF1 and ORF2, and was most closely related to the LC037415.1/Hu/GII.17/Kawasaki308 strain, forming a cluster with the strains detected in China, Japan and US at the nucleotide level (Figure 1). As observed in Figure 1, norovirus GII.17 strains genetically clustered in the phylogenetic tree, and all the non‐GII.17 sequences were located separately from the GII.17 cluster. Within the GII.17 cluster, the 2013/14 season strains were grouped separately from the GII.17 strains detected in 2014/15. HuzhouNS2014603 was clustered with the 2014/15 season strains. To further study the GII.17 cluster, a new tree based on the nucleotide sequence of capsid protein was built (Figure 2). Phylogenetic analysis based on full‐length sequences of the VP1 gene of norovirus GII.17 available from GenBank database showed that the sequences could be divided into three clusters, and cluster 3 further led to two subclusters (cluster 3a and cluster 3b). The HuzhouNS2014603 strains clustered together with strains collected in Hong Kong, China, Korea, JP, and United States from 2014 to 2015, cluster 3b. Other five strains collected in Japan and Taiwan from 2013 to 2014 formed cluster 3a, while earlier strains collected in 1978, 2002, 2005, 2008, and 2009 formed clusters 1 and 2. Although the novel GII.17 strains have emerged in 2013, two lineages could be distinguished easily on the tree.

### 3.3 Amino acid variation in the viral structural protein VP1

**Figure 3:**
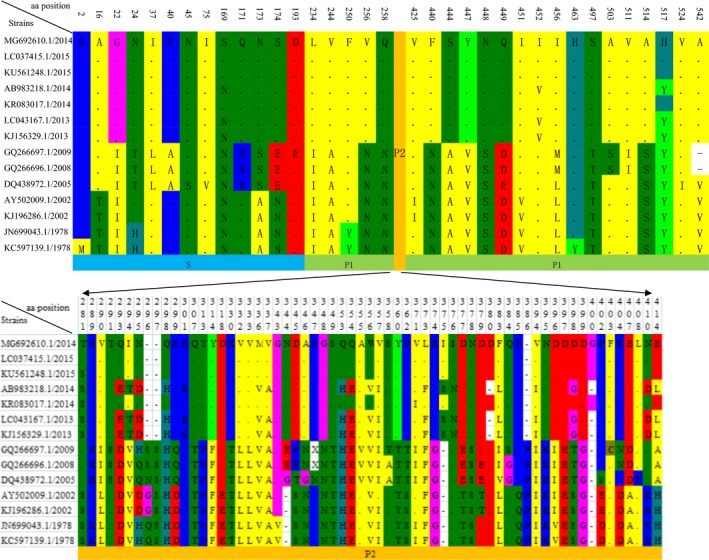
Amino acid substitutions in the VP1 sequence of norovirus GII.17 strains over time. Dashes indicate deletions/insertions of the amino acid residues. Amino acid numbering is based on the sequence of the Huzhou NS2014603 strain (MG692610.1). The accession numbers of norovirus strains used are as follows: LC037415.1, KU561248.1, AB983218.1, KR083017.1, LC043167.1, KJ156329.1, GQ266697.1, GQ266696.1, DQ438972.1, AY502009.1, KJ196286.1, JN699043.1, KC597139.1

The capsid protein (VP1) sequence of HuzhouNS2014603 was aligned with representative viruses of the other GII.17 clusters isolated from 1978 to 2015. Compared with other strains selected, there are 98 variable residues across the VP1 domain; the locations in the structure are shown in Figure 3. Among the 98 variable amino acids, 13 (13.3%) were observed in the shell domain and 22 (22.4%) in the P1domain; most of the substitutions and insertions were located in the P2 domain, account for 63 (64.3%), which contains the antigenic epitopes and host receptor binding domain (Donaldson, Lindesmith, Lobue, & Baric, 2010).

**Figure 4:**
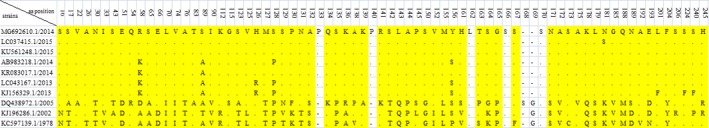
Amino acid substitutions in the VP2 sequence of norovirus GII.17 strains over time. Dashes indicate deletions/insertions of the amino acid residues. Amino acid numbering is based on the sequence of the HuzhouNS2014603 strain (MG692610.1). The accession numbers of norovirus strains used are as follows: LC037415.1, KU561248.1, AB983218.1, KR083017.1, LC043167.1, KJ156329.1, DQ438972.1, KJ196286.1, KC597139.1

As we can see most amino acid changes start from 2013, which is the time when the novel GII.17 first detected. The new GII.17s from 2013 to 2015 also can be divided into two branches, LC043167.1/2013, KJ156329.1/2013, and AB983218.1/2014 had the same amino acid, the other four had the same amino acid changes. The amino acid of the strains detected from 2005 to 2009 generally differed from the new GII.17 and the strains collected before 2005. So from Figure 4, the GII.17 mainly fall into three groups, 1978–2002, 2005–2009, 2013–2015, which is the same with phylogenetic tree.

### 3.4 Amino acid variation in the minor structural protein VP2

The minor structural protein VP2 sequence of HuzhouNS2014603 was aligned with the same representative viruses of the other GII.17 clusters isolated from 1978 to 2015 as VP1, except four strains which do not have the VP2 sequence. Besides the missing data from 2008–2009, the amino acid changes from 2013 mostly.

## 4 DISCUSSION

In contrast with genotype GII.4, GII.17 is an uncommon genotype, until recently, rarely detected in human cases (de Graaf et al., 2015). The norovirus GII.17 genotype was found in individual cases or in the waters of America, Korea, Thailand, Kenya, and China (Ferreira et al., 2012; Kittigul et al., 2010; Kiulia, Mans, Mwenda, & Taylor, 2014; Lee et al., 2013; Mans & Murray, 2014; Park et al., 2011; Wang et al., 2012), and was rarely detected in clinical diarrhea cases since 1978 (de Graaf et al., 2015). However, a novel lineage, termed GII.17_Kawasaki, emerged in October 2014 in Huzhou, Zhejiang, and replaced GII.4 to cause a significant increase in AGE outbreaks and also in sporadic AGE cases during the winter of 2014–2015 (Zhang et al., 2016). A similar prevalence was also observed in Guangdong, Hong Kong, Taiwan, and Japan during the same winter season (Fu et al., 2014; de Graaf et al., 2015; Lu et al., 2015; Matsushima et al., 2015; Taipei Times, 2015).

In the recent years, limitations in classifying viruses into genogroups based on partial nucleotide sequences have been a growing concern. Thus, Analysis of the entire sequence is a reliable approach to verify the characteristics of norovirus (Chhabra, Walimbe, & Chitambar, 2010; Lee, Jung, & Lee, 2012). Although the norovirus GII.17 genotype was found in recent outbreaks, only a few strains have had their full‐length genomes sequenced, especially in developing countries (Shen et al., 2011).

Phylogenetic analysis of the nearly full‐length sequences established a separate cluster for the norovirus GII.17 strains in a sister relationship with other known norovirus GII.17 strains and far from all the non‐GII.17 sequences. Although the novel GII.17 strains have emerged within the past 4 years, two lineages could be distinguished easily on the tree. The rapid evolution of the novel GII.17 strain may lead to escaping from the host immune response, affinity driven changes in tissue blood type antigens, and change the population susceptibility model (Chen et al., 2015; Debbink, Lindesmith, Donaldson, & Baric, 2012; Lochridge, Jutila, Graff, & Hardy, 2005). Based on the nearly full‐length genome, GII.17 have an evolutionary relationship with the GII.3/1998 strain and the GII.13/2006 strain, and presumably the two lineages of the novel GII.17 have their different evolutionary histories.

Analysis of VP1 protein sequences and VP2 protein sequencesreveals that most amino acid changes start from 2013. VP1 is further divided into the shell (S) domain and a protruding (P) domain. The S domain is highly conserved among different norovirus. The P domain is divided into the N‐terminal P1, C‐terminal P1, and P2 parts. The P2 domain was reported to be the most protruding and diverse among different norovirus groups (Prasad et al., 1999), indicating its critical function in interacting with host. Another study indicated that the GII.4 norovirus is continuously evolving through the alteration of the surface‐exposed receptor binding domain of the VP1 protein in response to human immune selection (Bull & White, 2011). Our findings indicated that the amino acid changes of the novel GII.17 strains over time are primarily located in the P2 subdomain, which is similar to the observations in GII.4 and other genotypes of the GII genogroup (Bull & White, 2011; de Graaf et al., 2015). norovirus GII.17 seems to have the identical mechanism in response to human immune selection with other GII genogroup.

There are two big replacements in the evolutionary history of GII.17, from the data on the full‐length VP1 gene available here. GII.17 genotype remained relatively stable for several decades prior to 2002. The replacements occurred during the period from 2002 to 2005 and the period from 2009 to 2013, when the amino acids of many important sites have changed at the same time. The former replacement did not cause a significant increase in AGE outbreaks and sporadic AGE cases. As we all know, the latter one cause a significant increase in AGE cases throughout the world. However, we still do not know which sites have played a key role in this event. Also the novel GII.17 seems to have two groups, 2013/2014 and 2014/2015. Some amino acids in the antigenic epitopes differed between 2013/2014 and 2014/2015. Were there new mutations again in 2014, or these two groups simply evolved from different ancestors as we said before?

## 5 CONCLUSIONS

This study confirms the HuzhouNS2014603 strain with nearly full‐length of 7556 nucleotides (nt) as norovirus GII.17 isolated in China. The results showed that norovirus circulating in the Huzhou area forming a cluster with the strains detected in other areas of China, Japan and United States at the nucleotide level and amino acid level. The capsid protein VP1 sequence of HuzhouNS2014603 was different from the other GII.17 clusters isolated from 1978 to 2009. The results of this study can be used in the study of the genetic evolution of the virus, the development of rapid diagnostic reagents and the design of vaccine.

## COMPETING INTERESTS

The authors declare that they have no competing interests.

## AUTHOR CONTRIBUTIONS

Conceived and designed the experiments: HJK. Performed the experiments: LPC XFW DSX. Analyzed the data: LPC LJ XFW. Contributed reagents/materials/analysis tools: LPC LJ XFW DSX. Wrote the paper: LPC.

## DECLARATIONS

Ethics Approval and Consent to Participate: Not applicable.

Consent for Publication: Not applicable.

Availability of Supporting Data: The data sets supporting the results of this article is included within the article.
